# Sociodemographic Predictors of Clinical Effectiveness, Therapeutic Program Engagement, and Device Usability for an In-Home Virtual Reality Program for Chronic Low Back Pain: Secondary Analysis of a Randomized Controlled Trial

**DOI:** 10.1089/jmxr.2023.0013

**Published:** 2024-04-18

**Authors:** Todd Maddox, Liesl Oldstone, Josh Sackman, Emily Judge, Roselani Maddox, Takisha Adair, Kelsey Ffrench, Charisse Y. Sparks, Beth D. Darnall

**Affiliations:** 1 AppliedVR, Inc., Van Nuys, California, USA.; 2 Inspire Medical Systems, Inc., Golden Valley, Minnesota, USA.; 3 Department of Anesthesiology, Perioperative and Pain Medicine, Stanford University School of Medicine, Palo Alto, California, USA.

**Keywords:** virtual reality, chronic low back pain, cognitive behavioral therapy

## Abstract

Health care inequities are well-established in chronic pain care. Immersive digital therapeutics may transcend such barriers by offering in-home access with no Wi-Fi connectivity required, and easy to use, gaze-based navigation, and therapeutic delivery. The objective of this report is to determine whether clinical effectiveness, therapeutic program engagement, and virtual reality (VR) device usability of an Food and Drug Administration (FDA)-authorized, proprietary skills-based VR program for chronic pain (i.e., RelieVRx^®^) are affected by key sociodemographic factors (age, gender, race/ethnicity, and socioeconomic status [SES]) often associated with reduced patient engagement and clinical effectiveness. We report a secondary analysis of a large (*n* = 1,093) randomized controlled trial that compared skills-based VR with active sham VR. The sample was demographically diverse with self-reported nonmalignant chronic low back pain ≥3 months duration with average pain intensity and pain interference ≥4/10. Data were collected from January 31, 2022, to October 31, 2022. The clinical effectiveness, therapeutic program engagement, and VR device usability of skills-based VR were generally unaffected by age (<65 vs. 65+ years), gender (male vs. female), race/ethnicity (White vs. Black vs. other), and SES (education and income), with a few exceptions (age difference for therapeutic program engagement; race/ethnicity difference for device usability). National calls for improved access to nonpharmacological pain care are addressed with this skills-based VR program, which is self-administered in-home, requires no Wi-Fi connectivity, uses gaze-based navigation, and requires on average 6 min/day.

TRIAL REGISTRATION: ClinicalTrials.gov NCT05263037

## Introduction

Chronic low back pain (cLBP) impacts approximately one-third of adults globally.^1^ With increasing efforts to curb opioid prescribing, effective, accessible nonpharmacological, behavioral treatments for cLBP are called for nationally by the U.S. Centers for Disease Control and Prevention (CDC) and U.S. Centers for Medicare and Medicaid Services (CMS).^1–4^ Pain education and cognitive behavior therapy (CBT) are recommended as first-line treatments given their low risk^5–8^ but barriers impede broad implementation. These include barriers to access because of the multisession, therapist-led nature of CBT, variability in effectiveness due in part to variation in the quality of therapists, and the inconsistent durability of effect up to 12 months after treatment.^9–14^

Digital devices, such as immersive virtual reality (VR), can deliver therapeutic content in a consistent, quality-controlled manner in the home, potentially addressing these shortcomings.^15,16^ A critically important additional advantage of VR over other digital technologies (e.g., smartphone mobile applications) is that the immersive nature of VR content broadly engages multiple centers in the brain in synchrony^16,17^ and can target pain processing brain regions known to be involved in cLBP^18–23^ that are responsive to treatments, such as CBT.^24–27^ Thus, therapeutic programs can be delivered in VR that combine pain education, CBT, diaphragmatic breathing, biofeedback, and mindfulness, which collectively help patients develop coping skills to address cLBP. The ease of use and the potential for repetition in VR allow these pain-coping skills to become habits that are long lasting and durable, all with a low-risk device accessible in-home.

Several VR-based chronic pain programs have been developed that show promise (see Refs. 28 and 29 for reviews), and one, a proprietary skills-based VR program for cLBP (called RelieVRx^®^), is FDA authorized.^30,31^ Despite the promise of VR as an immersive therapeutic for chronic pain, questions remain as to whether sociodemographic variabilities could impact patient engagement and contribute to the heterogeneity of treatment effectiveness.^32^ Several studies have found heterogeneity in the effectiveness of digital therapeutics as a function of age, gender, race/ethnicity, and socioeconomic status (SES), with patients who are older, females, non-White, or of low SES being disadvantaged.^32–35^ Critically, key factors identified to address these inequities are broad availability (e.g., no Wi-Fi necessary) and ease of use (e.g., minimal time commitment, in-home administration, and simple navigation). New digital health technologies should specifically evaluate these factors within the context of key sociodemographic factors, which is the goal of this report.

A recent double-blinded, randomized placebo-controlled trial^30^ (see also Ref. 31) compared the 56-session, in-home self-administered skills-based VR program to active sham VR in a large sample (*n* = 1,093) of adults with cLBP that was demographically diverse and clinically severe (30+% non-White, 20% high school or less, baseline pain intensity = 6.6/10; baseline pain interference = 6.2/10, baseline disability = severe/completely disabled [based on the Oswestry Disability Index]; baseline sleep disturbance = moderate/severe [based on the PROMIS Sleep Disturbance survey]). Clinically meaningful reductions^36,37^ in pain intensity (2.0) and pain interference (2.3) were observed at the end of treatment for skills-based VR that were significantly larger than for sham. The VR device obtained an A+ rating on the System Usability Scale,^38^ and participants completed an average of 37.6 of the 56 sessions.^30^

The current study presents a secondary analysis of this large, demographically diverse, and clinically severe sample to determine whether technology-based challenges exist for sociodemographic subgroups. Specifically, we examine the patient-reported clinical effectiveness, objectively determined therapeutic program engagement, and patient-reported VR device usability ratings for patients who received the skills-based VR program as a function of the sociodemographic factors of age, gender, race/ethnicity, income, and education levels (SES) (for completeness, the same analyses were conducted for the sham VR group). Because the sample size is large,^30^ we have the statistical power necessary to conduct meaningful subgroup analyses.

## Methods

### Study design, participants, and randomization

Full methods for the clinical trial are published, including the CONSORT diagram.^30^ This was a fully decentralized randomized controlled trial comparing skills-based VR with an active sham VR control. Individuals with self-reported cLBP (>3 months and average pain intensity and interference of >4 for the past month on a 0–10 pain rating scale; Brief Pain Inventory [BPI]^39^) were recruited through online advertisements, chronic pain organizations, and pain clinics. Once consented, cLBP participants were randomized at a 1:1 ratio and allocated to skills-based VR or sham VR treatment groups. The skills-based VR program offers a fixed sequence of 56 VR sessions (average duration of 6 min) that incorporate evidence-based self-regulatory skills used in CBT for chronic pain, mindfulness, and pain neuroscience education. Sham VR offers a fixed sequence of 56 nonimmersive, 2D nature videos overlaid with neutral music. The study protocol was approved by the WCG Institutional Review Board (Puyallup, WA) in December 2021 and followed the Consolidated Standards of Reporting Trials (CONSORT) reporting guidelines. Written informed consent was obtained before enrollment. Study data were collected from January 31, 2022, to October 31, 2022.

The focus of this report is to determine whether clinical effectiveness, therapeutic program engagement, and VR program usability for the skills-based VR group are affected by the sociodemographic factors of age (<65 vs. 65+ years), gender (male vs. female), race/ethnicity (White vs. Black vs. other^1^), and SES (low vs. high). An age cutoff of 65 years was selected because of the importance of that age at CMS. Only two nonbinary participants were enrolled in the trial so a dichotomy of male versus female was chosen. Sample size considerations did not allow a more granular breakdown of race/ethnicity or SES. For completeness, the same analyses are conducted on the sham VR group.

### Patient demographics, patient-reported outcomes, and study group interventions

Several demographics were collected before the initiation of assigned treatment. Most relevant to the current report were age, gender, race/ethnicity, and annual household income and education. In line with previous work from our lab,^40^ the latter two were used to define a binary SES factor with high SES being associated with participants with post high school education and an annual household income greater than $60,000. Several patient-reported outcomes were collected before and immediately following the treatment. The BPI^39^ addresses clinical effectiveness and measures average pain intensity and pain interference over the last 24 h using a 0–10 numeric pain rating scale.^39^ The System Usability Scale was administered at the end of treatment^38^ which defines usability on a 0–100 scale and an associated letter grading system and measures VR device usability. Finally, therapeutic program engagement was measured by the number of VR experiences completed during the 56-session therapy that was objectively determined through device download when returned to the manufacturing facility.

### Statistical analysis

All data are expressed as mean ± standard deviation. Clinical effectiveness measures include reductions in BPI pain intensity and pain interference from baseline to end of treatment, and responder rates are defined as the percentage of participants who achieved a clinically meaningful 2.0-point reduction from baseline to end of treatment in pain intensity, pain interference, or both. Therapeutic program engagement is quantified as the number of VR experiences completed during the 56-session intervention, and the usability is measured with the System Usability Scale. The analytic approach is as follows. First, we conducted three sets of linear regression analyses. One with pain intensity reduction, second with pain interference reduction, and a third with responder rate as the outcome measure. Within each set, the predictor variables were treatment (skills-based VR vs. sham VR) and the relevant sociodemographic subgroup (age, gender, race/ethnicity, or SES). Next, we conducted univariate general linear model analyses of the skills-based VR data with models being applied separately to each of the three clinical effectiveness measures, as well as the therapeutic program engagement and VR device usability measures with each of the four sociodemographic factors as predictors (a total of 20 analyses). Because of concerns with multiple comparisons, we used the Bonferroni-adjusted significance level of 0.0025 (adjusted significance level = 0.05/20 = 0.0025). Finally, and for completeness, we repeated these analyses for the sham VR data.

## Results

Table 1 displays the *clinical effectiveness* results: average pain intensity reduction, average pain interference reduction, percentage of responders, and the average pain reductions for responders by age (65+ vs. <65 years), gender [male vs. female (two nonbinary participants were excluded from the analysis)], race/ethnicity (White vs. Black vs. other), and SES (low vs. high). Table 1 also displays the average *therapeutic program engagement* and the average *VR device usability*. Table 2 uses the same format to display the sham VR results.

**Table 1.** Average ± Standard Deviation (and Effect Size) for RelieVRx^®^ Clinical Effectiveness, Therapeutic Program Engagement, and Device Usability by Sociodemographic Factor

	Age		Gender		Race/ethnicity			SES^a^	
	<65	65+	Female	Male	White	Black	Other	Low SES	High SES
Modified intent-to-treat, *n*	449	87	411	124	370	81	85	332	204
Clinical effectiveness									
Pain intensity reduction									
Mean ± SD	1.9 ± 2.0	1.9 ± 1.9	2.0 ± 1.9	1.7 ± 1.9	1.8 ± 1.9	2.1 ± 2.1	2.2 ± 2.0	1.9 ± 2.0	2.0 ± 1.8
Effect size	<0.001		0.005		0.007			<0.001	
Pain interference reduction									
Mean ± SD	2.2 ± 2.0	2.1 ± 1.7	2.3 ± 2.0	1.8 ± 1.9	2.2 ± 1.9	2.3 ± 2.1	2.3 ± 2.1	2.2 ± 2.1	2.2 ± 1.8
Effect size	<0.001		0.014		0.001			<0.001	
% Responder^b^									
Mean	58.3	58.2	59.8	52.3	55.8	61.3	65.8	59.4	56.5
Effect size	<0.001		0.004		0.006			0.001	
Average reduction for responders									
Mean ± SD	3.6 ± 1.3	3.4 ± 1.3	3.6 ± 1.3	3.3 ± 1.3	3.5 ± 1.3	3.7 ± 1.5	3.6 ± 1.2	3.5 ± 1.4	3.5 ± 1.1
Therapeutic program engagement									
Average RelieVRx^®^ sessions completed									
Mean ± SD	37.6^*^ ±16.8	47.2^*^ ± 11.2	39.2 ± 16.0	38.4 ± 16.0	40.0 ± 15.2	34.4 ± 19.2	39.2 ± 14.4	40.0 ± 16.0	37.6 ± 16.0
Effect size	0.052		0.002		0.013			0.002	
VR device usability									
Average system usability									
Mean ± SD	91.6 ± 10.7	91.7 ± 9.5	91.8 ± 10.6	91.0 ± 10.3	92.8^*^ ± 9.2	88.5^*^ ± 13.1	90.0^*^ ± 12.3	91.5 ± 11.1	91.9 ± 9.4
Effect size	<0.001		0.001		0.026			<0.001	

SES, socioeconomic status; SD, standard deviation; VR, virtual reality.

a High SES is defined as those with more than a high school education and greater than $60,000/year household income.

b Responder is defined as any participant with at least a two-point reduction in pain intensity, pain interference, or both.

* *p* < 0.0025.

**Table 2.** Average ± Standard Deviation (and Effect Size) for Sham VR Clinical Effectiveness, Therapeutic Program Engagement, and Device Usability by Sociodemographic Factor

	Age		Gender		Race/ethnicity			SES^a^	
	<65	65+	Female	Male	White	Black	Other	Low SES	High SES
Modified intent-to-treat, *n*	434	97	362	169	351	95	85	334	197
Clinical effectiveness									
Pain intensity reduction									
Mean ± SD	1.4 ± 1.9	1.7 ± 1.8	1.5 ± 1.9	1.4 ± 1.8	1.5 ± 1.9	1.6 ± 1.9	1.3 ± 1.5	1.5 ± 1.8	1.5 ± 1.9
Effect size	0.002		0.001		0.004			<0.001	
Pain interference reduction									
Mean ± SD	1.6 ± 1.9	1.7 ± 1.7	1.7 ± 1.9	1.5 ± 1.8	1.7 ± 1.9	1.7 ± 1.9	1.5 ± 1.7	1.7 ± 1.9	1.5 ± 1.8
Effect size	<0.001		0.002		0.001			0.004	
% Responder^b^									
Mean	46.3	52.9	49.2	43.5	48.1	48.8	43.4	47.7	47.2
Effect size	0.003		0.002		0.001			<0.001	
Average reduction for responders									
Mean± SD	3.3 ± 1.1	3.2 ± 1.0	3.3 ± 1.1	3.3 ± 1.2	3.3 ± 1.1	3.3 ± 1.2	3.2 ± 0.9	3.3 ± 1.1	3.2 ± 1.1
Therapeutic program engagement									
Average RelieVRx^®^ sessions completed									
Mean ± SD	29.6^*^±19.2	37.6^*^±16.0	31.2 ± 18.4	31.2 ± 20.0	33.6^*^±18.4	25.6^*^±19.2	27.2^*^±19.2	30.4 ± 20.0	31.2 ± 19.2
Effect size	0.022		<0.001		0.031			0.001	
VR device usability									
Average system usability									
Mean ± SD	86.6 ± 14.0	83.6 ± 15.5	87.0 ± 13.9	83.8 ± 15.1	86.3 ± 13.9	84.1 ± 17.1	87.0 ± 12.6	86.0 ± 13.7	86.0 ± 15.4
Effect size	0.007		0.011		0.004			<0.001	

SES, socioeconomic status; SD, standard deviation; VR, virtual reality.

a Low SES is defined as those with a high school education or less or $60,000/year or less income.

b Responder is defined as any participant with at least a two-point reduction in pain intensity, pain interference, or both.

* *p* < 0.0025.

### Clinical effectiveness

For all 12 linear regression analyses (three effectiveness outcome measures and four sociodemographic predictors) that included a treatment group and a sociodemographic factor as predictors, the treatment group effect was statistically significant (all *p* values <0.001) and the sociodemographic factor was not. Thus, we examine sociodemographic factors separately by treatment. None of the four sociodemographic factors was a significant predictor of the pain intensity reduction, pain interference reduction, or responder rate for skills-based VR (Table 1) or sham VR (Table 2).

### Therapeutic program engagement

Age was a significant predictor of skills-based VR and sham VR program engagement. Interestingly, in both cases, participants aged 65 years or older completed significantly more experiences. In addition, race/ethnicity was a predictor for sham VR (but not skills-based VR) with White and other participants completing more experiences than Black participants.

### VR device usability

Only race/ethnicity was a statistically significant predictor of skills-based VR system usability, with White and other participants rating higher device usability than Black participants. Even so, the average rating was A+ for all groups. None of the four sociodemographic factors was a significant predictor of sham VR usability.

## Discussion

Barriers to health care impede treatment equity and access across several sociodemographic groups, and this is well-known in pain care.^9,32^ These barriers can involve challenges with therapeutic access (e.g., limited access to trained therapists or in-home therapies) and challenges with therapeutic delivery (e.g., the need to drive to receive therapy or the lack of digital therapies in multiple native languages). Packaging a safe and effective chronic pain curriculum into a VR headset that does not require Wi-Fi and can be sent to the home can overcome access barriers, and simplifying the user interface with gaze-based navigation can make delivery of the therapy as simple as pressing the power button and placing the headset comfortably on the head.

We examined the influence of sociodemographic factors on patient-reported clinical effectiveness, objective therapeutic program engagement, and patient-reported VR device usability ratings for an FDA-authorized, in-home, self-administered skills-based VR program that addresses many of these barriers to access and delivery, in data from a recently completed randomized controlled trial with a large demographically diverse and clinically severe sample of cLBP participants.^30^

Most importantly, clinical effectiveness was strong and invariant across age, gender, race/ethnicity, and socioeconomic subgroups for skills-based VR (and sham VR). Therapeutic program engagement was influenced only by age, and contrary to the common belief, older adults aged 65+ years showed greater engagement than adults aged <65 years for both skills-based VR and sham VR. Skills-based VR device usability was high and invariant across age, gender, race/ethnicity, and socioeconomic subgroups for skills-based VR, and only race/ethnicity yielded an effect for sham VR with White and other participants showing the highest usability. Even so, across sociodemographic (and treatment) groups usability received an A+ rating.

The finding that clinical effectiveness, therapeutic program engagement, and VR device usability of the skills-based VR program were generally invariant to the sociodemographic factors of age, gender, race/ethnicity, and SES is important. It helps mitigate some of the uncertainty about the degree to which underrepresented populations may benefit from home-based digital therapeutics,^32–35^ especially given the fact that some underrepresented groups have undertreated pain.^41–45^

The strong clinical effectiveness of the skills-based VR program that is invariant across sociodemographic factors may be because of (1) the participant-reported ease of use of the VR device; (2) the minimal time commitment of 6 min per day on average; and (3) the in-home, on-demand convenience of the skills-based VR device that does not require Wi-Fi connectivity. Because these factors are unique to the hardware and software specific to this particular skills-based VR program, these results may not be generalizable to other VR-delivered therapeutic programs. Despite these strengths, one major limitation of the skills-based VR program is that it is currently available only in English. Expansion to other languages such as Spanish is a priority to truly address barriers to access.

### Limitations

Limitations to consider when evaluating the study results include (1) a reliance on participant-reported data with no objective verification of the sociodemographics or data on health care utilization, (2) the use of a single definition on SES, (3) inability to conduct more granular analyses on race/ethnicity, (4) self-reported cLBP that was not confirmed by healthcare professionals, and (5) a focus on cLBP without examining comorbid or other chronic pain conditions.

## Conclusions

Community-based clinicians struggle to find in-home, low-risk, nonpharmacologic options to treat cLBP whose effectiveness is robust across several important sociodemographic factors such as age, gender, race/ethnicity, and SES. The promising results of this study suggest that the skills-based VR program can provide consistent, quality-controlled, easily accessible cLBP treatment that is effective and robust across sociodemographic factors.

## Summary

Data suggest that a skills-based VR program for chronic pain’s clinical effectiveness, therapeutic engagement, and device usability is invariant across key sociodemographic factors and thus can help transcend pain care disparities via in-home treatment.

## Data Availability

A data dictionary and de-identified participant data will be made available after publication and upon approved request of a detailed meta-analytic study proposal. Requests should be made to the corresponding author along with a study proposal and a signed data access agreement.

## Abbreviations Used

VR: virtual reality
cLBP: chronic low back pain
CDC: US Centers for Disease Control and Prevention
CMS: US Centers for Medicare and Medicaid Services
CBT: cognitive behavioral therapy
SES: socioeconomic status
CONSORT: Consolidated Standards of Reporting Trials
FDA: Food and Drug Administration

## Notes

1 Because the race/ethnicity sample sizes for American Indian/Alaska Native (*n* = 4), Asian/Pacific Islander (*n* = 12), Hispanic/Latin (*n* = 18), and multiracial (*n* = 51) were small, these participants were assigned to the non-White, non-Black, and “other” category.
